# When the Air Gets Thin, and the Blood Gets Thick: Choroidal Hemangioma in High-Altitude Polycythemia

**DOI:** 10.7759/cureus.103419

**Published:** 2026-02-11

**Authors:** Sonali V Kumar, Ashok Meshram, Vinay Kumar, Alok Sati, Natasha V Kumar

**Affiliations:** 1 Ophthalmology, Command Hospital (Eastern Command) Kolkata, Kolkata, IND; 2 Hematology and Oncology, Command Hospital (Eastern Command) Kolkata, Kolkata, IND; 3 Anatomy, JIS School of Medical Science and Research, Kolkata, IND; 4 Ophthalmology, Army Hospital Research and Referral, Delhi, IND; 5 Medicine, Sri Devaraj Urs Medical College, Kolar, IND

**Keywords:** choroidal hemangioma, choroidal vascular tumors, high-altitude polycythemia, hyperviscosity, hypoxia, retina

## Abstract

Choroidal hemangioma is a rare benign vascular tumor that can cause visual impairment due to associated subretinal fluid. Systemic factors influencing its presentation and activity are not well-understood. We report a unique case of choroidal hemangioma diagnosed in a patient who developed high-altitude polycythemia, highlighting the possible role of systemic hypoxia and blood hyperviscosity in aggravating choroidal vascular tumors. A 36-year-old male soldier, posted at an altitude of approximately 16,300 feet, presented with painless diminution of vision in the right eye. Fundus examination revealed an orange-red, elevated lesion located superior to the optic disc, approximately two disc diameters in size, with subretinal fluid extending to the fovea, suggestive of choroidal hemangioma. Hematological work-up revealed hemoglobin 18.8 g/dL and hematocrit 60.6%, consistent with high-altitude polycythemia. The serum erythropoietin level was normal, while the JAK 2 mutation and antinuclear antibody (ANA) were negative. Lipid profile demonstrated hypertriglyceridemia (525 mg/dL) and elevated very low-density lipoprotein (VLDL) (105 mg/dL). The patient underwent three sessions of therapeutic phlebotomy, following which the hemoglobin decreased to 16.6 g/dL. The patient also received a single intravitreal Eylea (aflibercept) injection, following which the subretinal fluid subsided significantly. The patient was subsequently advised to avoid future high-altitude exposure after his hematocrit normalized and visual acuity improved. Awareness of systemic contributory factors is important in the evaluation and management of choroidal vascular tumors. Choroidal hemangioma in the setting of high-altitude polycythemia is rare. Chronic hypoxia-induced hyperviscosity may contribute to vascular dilation and permeability changes within the choroid. Recognition of this association is crucial for timely diagnosis and effective management of choroidal hemangioma to preserve vision.

## Introduction

Choroidal hemangioma is a benign vascular hamartoma of the choroid, composed of cavernous vascular channels lined by endothelium and supported by thin connective tissue [1]. It may occur in two forms: diffuse, often associated with Sturge-Weber syndrome (SWS), and circumscribed, which is typically solitary and idiopathic [2]. The circumscribed variant usually presents in adults between the third and fifth decades of life [3,4]. Clinical symptoms arise due to serous retinal detachment overlying the vascular mass, leading to decreased vision, metamorphopsia, or photopsia [4]. The lesion’s characteristic orange-red color and distinct angiographic features aid in diagnosis [3,4]. Although the lesion is usually stable, its behavior may be influenced by systemic or environmental factors that alter ocular hemodynamics. Changes in blood viscosity, oxygenation, and choroidal perfusion can modulate fluid dynamics within the lesion and surrounding retina.

SWS is a rare neuro-oculo-cutaneous disorder characterized by a facial port-wine stain, leptomeningeal angioma, and ocular vascular anomalies, including choroidal hemangioma and glaucoma. The vascular malformations in SWS arise due to defective regression of embryonic vascular plexuses [2-4]. Diagnosis is primarily clinical, supported by neuroimaging demonstrating leptomeningeal vascular malformations.

High-altitude polycythemia (HAPC) represents a physiologic response to chronic hypobaric hypoxia, with increased erythropoietin secretion and compensatory erythrocytosis. This adaptive process improves oxygen transport but also raises blood viscosity, predisposing to vascular congestion and tissue hypoxia, which can affect choroidal and retinal microcirculation [5].

High-altitude exposure is known to produce vascular changes such as venous dilation, microhemorrhages, and papilledema, collectively termed high-altitude retinopathy. However, choroidal vascular involvement in this context has rarely been documented [6].

While several systemic effects of HAPC are well-recognized, its influence on ocular vascular lesions has not been reported. Herein, we report a unique case of circumscribed choroidal hemangioma associated with SWS in a soldier stationed at high altitude, where systemic polycythemia likely aggravated choroidal exudation. This case emphasizes the potential role of hypoxia and hyperviscosity in unmasking or exacerbating choroidal vascular lesions and highlights successful management with anti-VEGF therapy and systemic correction. To our knowledge, such a coexistence has rarely been documented in the literature.

## Case presentation

A 36-year-old male patient soldier presented with painless, progressive diminution of vision associated with metamorphopsia in his right eye for one month. He described distortion of central vision, particularly while reading or focusing on straight lines. The symptoms began during his posting at a high-altitude region (16,300 feet), where he had been deployed for nearly one year as part of his military service. There was no history of ocular pain, photopsia, trauma, or previous ocular disease or systemic disease such as hypertension or diabetes mellitus. He denied systemic symptoms such as headache, dizziness, or breathlessness. He initially reported to a high-altitude medical center with similar complaints. He was subsequently transferred to our tertiary care center located in the plains for further ophthalmic assessment.

**Figure 1:**
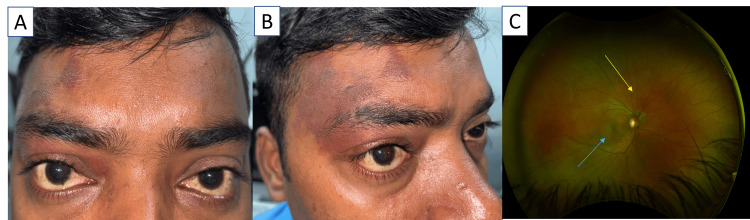
A & B: Clinical photograph depicting violaceous pigmented lesions involving the right forehead and periocular region. C: Fundus photograph showing a dome-shaped lesion, orange-red choroidal lesion located superior to the optic disc (yellow arrow), associated with serous retinal detachment reaching the macula and foveal region (blue arrow).

On general examination, the patient had flat, violaceous pigmentary lesions over the right forehead and periocular region, corresponding to the ophthalmic (V1) division of the trigeminal nerve, consistent with a port-wine stain (Figures 1A, 1B). There were no lesions elsewhere on the body. Neurological examination was normal, and there was no limb hypertrophy or meningeal signs.

Ocular examination revealed best corrected visual acuity (BCVA) of 6/60 in the right eye and 6/6 in the left eye. Pupils were equal and reactive, with no relative afferent pupillary defect. Extraocular movements were full and painless in all directions. The anterior segment was normal, with a clear cornea, quiet anterior chamber, and clear lens. Intraocular pressure was 18 mm in the right eye and 16 mm in the left eye by Goldmann applanation tonometry. Fundus examination of the right eye revealed a well-circumscribed, orange-red, dome-shaped choroidal lesion located at the optic disc, measuring approximately two disc diameters (DD) in horizontal extent. The surface vessels were slightly dilated and tortuous. A serous retinal detachment was observed extending toward the macula and involving the fovea, explaining the metamorphopsia (Figure 1C). The optic disc was healthy, and the remainder of the retina was unremarkable. The left eye fundus was normal.

**Figure 2:**
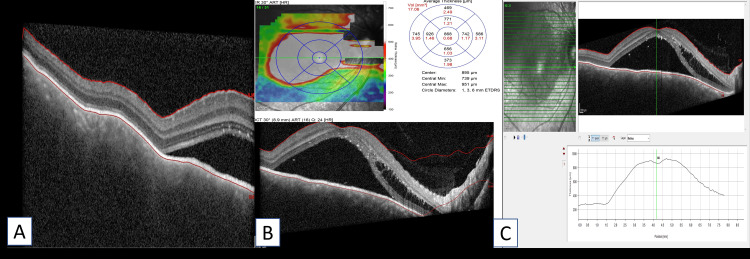
A-C: Optical coherence tomography showed a smooth, dome-shaped choroidal elevation with associated subretinal fluid at the fovea.

**Figure 3:**
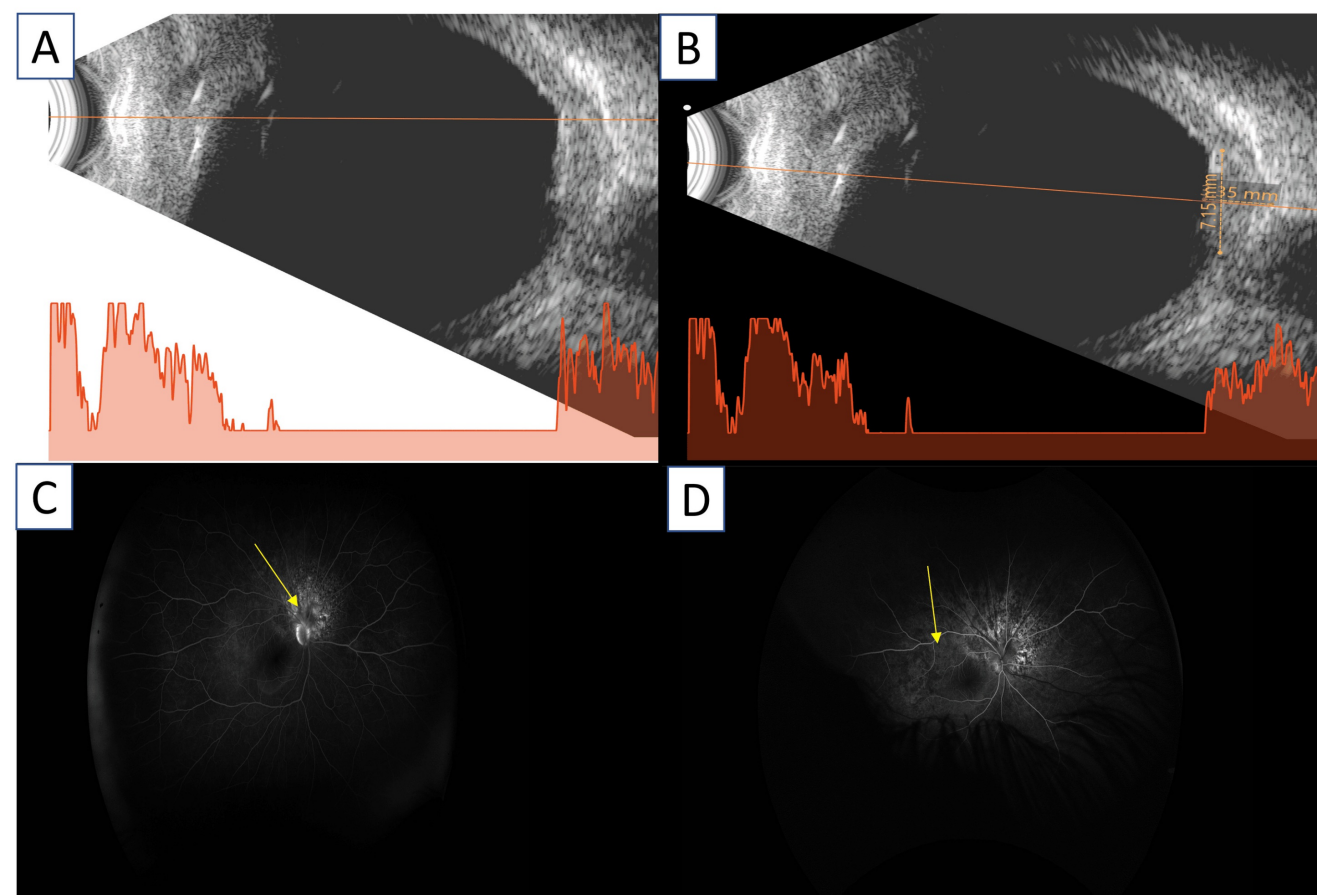
A & B: B-scan ultrasonography demonstrated an acoustically solid, homogenous intraocular lesion measuring 6.4 mm at its base and 2.35 mm in thickness. C & D: Fundus fluorescein angiogram depicting early hyperfluorescence of the lesion with increasing intensity in the mid-phase and late leakage of dye (yellow arrow).

Optical coherence tomography (OCT) demonstrated a smooth, dome-shaped choroidal elevation corresponding to the lesion, with neurosensory retinal detachment extending to the fovea. Outer retinal layers showed mild disruption at the detached retina (Figures 2A-2C). Central macular thickness was 861 µm. B-scan ultrasonography showed a homogenous, acoustically solid lesion measuring 6.4 mm in basal diameter and 2.35 mm in thickness, without calcification or extrascleral extension (Figures 3A, 3B). Fundus fluorescein angiography (FFA) showed early hyperfluorescence of the lesion with progressive hyperfluorescence in the mid phase and late leakage of dye into the surrounding subretinal space, suggestive of active choroidal hemangioma (Figures 3C, 3D).

Based on these clinical imaging features, a diagnosis of circumscribed choroidal hemangioma associated with SWS was established.

The patient was referred to a medical specialist for systemic assessment to rule out further involvement associated with SWS. Neurological examination was normal, and MRI of the brain revealed no evidence of leptomeningeal angioma or cerebral atrophy, confirming an ocular-limited form of the syndrome. Laboratory investigations showed hemoglobin 18.8 g/dL, hematocrit 60.6%, RBC count 6.67 million/µL, and serum erythropoietin 2.8 mU/mL. Lipid profile showed triglyceride levels of 525 mg/dL and VLDL levels of 105 mg/dL. Renal, hepatic, and thyroid levels were within normal limits. The JAK2 gene mutation was not detected by the PCR technique. ANA was also found to be negative (Table 1).

**Table 1:** Key laboratory findings

Investigation	Result	Normal range	Interpretation
Hemoglobin	18.8 g/dl	13.5 to 17.5 g/dL	Polycythaemia
Hematocrit	60.6%	41%-50%	Polycythaemia
Red blood cell count	6.67 million/µL	4.7-6.1 million/µL	Polycythaemia
Serum erythropoietin level	2.8 mU/mL	2.6-18.5 mU/mL	Normal
Triglycerides level	525 mg/dL	<150 mg/dL	Hypertriglyceridemia
Very low-density lipoprotein (VLDL) level	105 mg/dL	2- 30 mg/dL	Dyslipidaemia
Janus Kinase 2 (JAK2) gene mutation	Not detected	-	Ruled out primary polycythaemia
Antinuclear antibody (ANA)	Negative	-	Ruled out autoimmune disorder

Routine blood investigation revealed elevated hemoglobin and hematocrit, suggestive of secondary polycythemia due to chronic hypoxia at high altitude. The absence of JAK2 mutation and negative ANA, combined with documented hypoxemia and normal erythropoietin level, also confirmed secondary HAPC due to chronic hypoxia, rather than a primary or autoimmune disorder.

The patient underwent three sessions of therapeutic phlebotomy over two weeks, following which hemoglobin decreased to 16.6 g/dL and hematocrit normalized to 50%. He was also started on Tab Ecosprin 75 mg, Tab Atorvastatin 10 mg, and Tab Fenofibrate 200 mg at bedtime for one month. He was also advised adequate hydration, avoidance of re-exposure to high altitude, and regular follow-up with hematologic and lipid monitoring.

Considering the foveal involvement and symptomatic metamorphopsia, the patient received a single intravitreal injection of aflibercept (Eylea, 2 mg/.05ml) under aseptic precautions.

**Figure 4:**
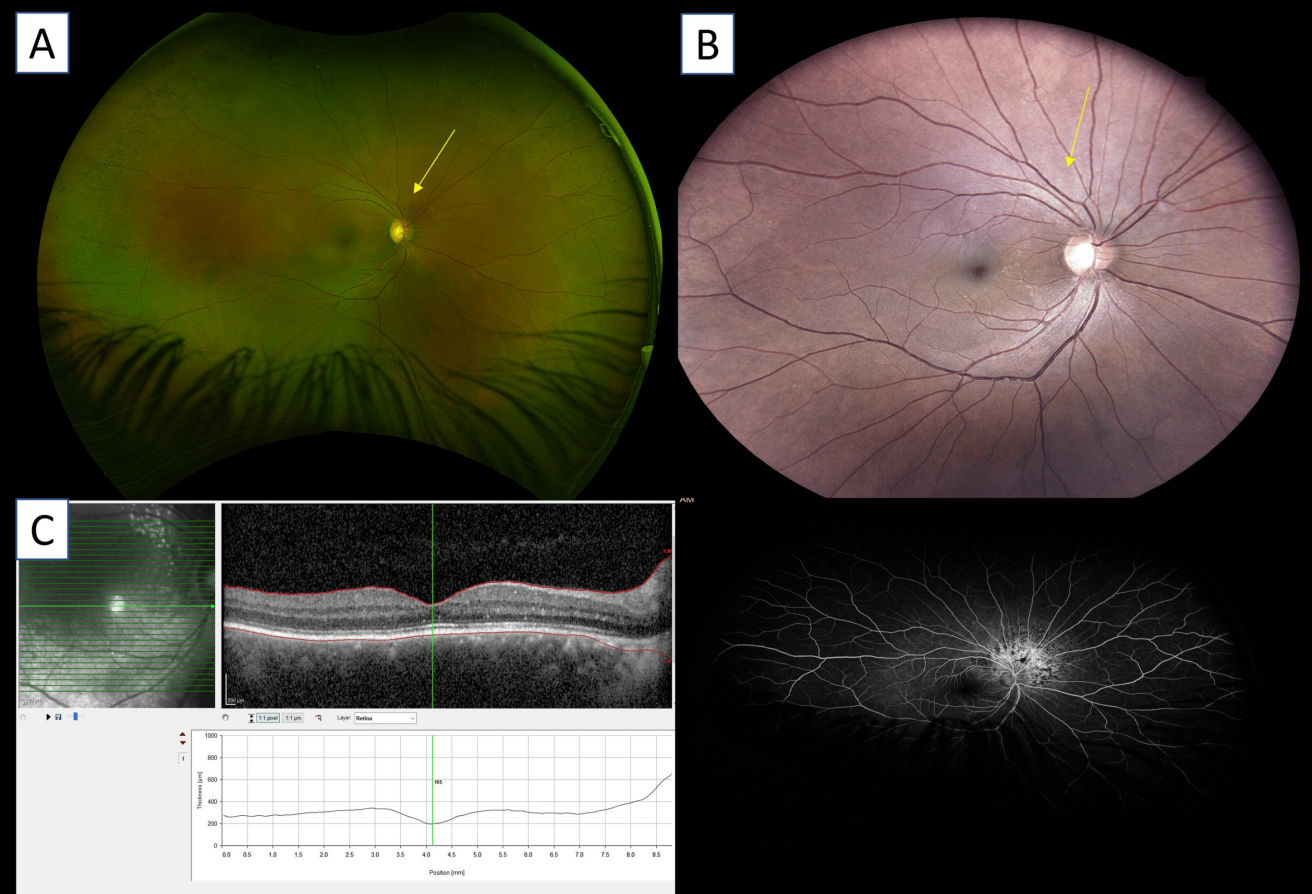
A & B: Color fundus photograph showing a regressed, inactive choroidal lesion superior to the optic disc one month after intravitreal aflibercept injection (yellow arrow). C: Optical coherence tomography showing the complete absorption of subretinal fluid with the recovery of normal foveal configuration one month following intravitreal aflibercept therapy. D: Fundus fluorescein angiogram revealing diminished hyperflurorescence and lack of late leakage, suggestive of an inactive lesion one month post aflibercept injection.

At four weeks, BCVA further improved to 6/9 with regression of the tumor (Figures 4A, 4B), complete resolution of subretinal fluid, and restoration of normal foveal contour on OCT (Figure 4C). FFA was repeated, which showed a previously active choroidal hemangioma showing reduced hyperfluorescence with the absence of late leakage, consistent with an inactive lesion following anti-VEGF therapy (Figure 4D). No abnormal pooling, leakage, or blocked fluorescence was found in the left eye.

At the six- month follow-up, the lesion remained stable with no recurrence of exudation or fluid. Systemic parameters remained normal (Hb 13 g/dl, Hematocrit 40.2%), and the lipid profile showed improvement with ongoing therapy. The periocular port-wine stain remained unchanged.

## Discussion

This case illustrates a unique interplay between ocular vascular pathology and systemic hematologic alteration. The patient presented with visual disturbances attributable to serous retinal detachment secondary to choroidal hemangioma, with the unusual coexistence of secondary high-altitude polycythemia and cutaneous angiomatous lesions of SWS.

While choroidal hemangioma is typically a benign, congenital vascular lesion, its clinical behavior can be influenced by systemic or local hemodynamic factors. The coexistence of chronic hypoxia-induced polycythemia may have contributed to increased blood viscosity and choroidal venous congestion, thereby aggravating vascular leakage from the pre-existing hemangioma. This could explain the onset of metamorphopsia and subfoveal fluid accumulation while the patient was stationed at high altitude.

The normal erythropoietin level and absence of JAK2 mutation ruled out primary polycythemia, confirming a secondary adaptive erythrocytosis. The hypoxic environment of high altitude, with reduced arterial oxygen saturation, was the most likely trigger for increased red cell mass. The accompanying dyslipidemia, with marked hypertriglyceridemia and elevated VLDL, may have further compounded vascular dysfunction and leakage tendency. Such multifactorial vascular stress can significantly influence the clinical course of ocular vascular lesions like choroidal hemangioma.

An additional noteworthy aspect of this case was the rapid anatomical and visual improvement following a single intravitreal aflibercept injection. While anti-VEGF agents have been used as adjunctive therapy in selected cases of choroidal hemangioma, their role in the setting of secondary high altitude polycythemia and cutaneous vascular lesions suggestive of a Sturge-Weber spectrum has not been previously described. Anti-VEGF agents act by reducing vascular permeability and downregulating angiogenic signaling, thereby controlling exudation. The choice of aflibercept was particularly advantageous in this setting due to its broader VEGF and placental growth factor blockade [7].

Among the various treatment options described for choroidal hemangioma, including photodynamic therapy (PDT), laser photocoagulation, transpupillary thermotherapy, and radiotherapy, anti-VEGF therapy offers a minimally invasive, effective alternative, especially in patients with systemic hyperviscosity or vascular comorbidities, where laser-based or radiotherapy modalities may be less desirable [4,8-10]. The outcome in this patient supports the growing evidence for the role of anti-VEGF monotherapy in selected hemangioma cases with exudative detachment.

In addition to local ocular therapy, systemic management played a complementary role in this case. Secondary high-altitude polycythemia is associated with increased blood viscosity and altered microvascular flow, which may influence choroidal hemodynamics and exacerbate exudation from vascular tumors such as choroidal hemangioma. Therapeutic phlebotomy effectively reduced hemoglobin and hematocrit levels, potentially mitigating hyperviscosity-related choroidal congestion. Concurrent management of dyslipidemia with statin-fibrate therapy further addressed systemic vascular risk factors. While these interventions cannot be directly linked to tumor regression, they provide important pathophysiological context and highlight the relevance of systemic optimization in the overall management of such patients.

Although associations of choroidal hemangioma with polypoidal choroidal vasculopathy, branch retinal vein occlusion, and pregnancy have been previously reported, its coexistence with high-altitude polycythemia has not been described in the literature so far [11,12].

This case brings attention to an unusual but clinically meaningful association, that is, high-altitude exposure, as a potential exacerbating factor in vascular lesions. In susceptible individuals, altitude-induced hematologic changes may unmask or worsen pre-existing vascular anomalies, underscoring the importance of systemic evaluation in any atypical or recurrent choroidal exudation.

## Conclusions

This report highlights the rare coexistence of choroidal hemangioma with cutaneous vascular lesions suggestive of a Sturge-Weber spectrum and secondary high-altitude polycythemia. Visual decline was attributable to serous retinal detachment secondary to the choroidal hemangioma; however, chronic hypoxia and blood hyperviscosity may have precipitated or exacerbated tumor-related exudation, influencing the clinical presentation. Early recognition, targeted systemic correction through phlebotomy and lipid control, and intravitreal anti-VEGF therapy resulted in complete recovery and vision improvement. The case underscores the need for comprehensive systemic assessment in patients with exudative choroidal lesions, particularly in those with vascular cutaneous features or prolonged high-altitude exposure. Awareness of such rare but clinically relevant interactions can aid timely diagnosis, prevent recurrence, and preserve vision through a coordinated multidisciplinary approach.
